# Research on the utilization of community health services and its determinants among older adults: a cross-sectional study in Liuzhou City

**DOI:** 10.3389/fpubh.2026.1885041

**Published:** 2026-09-07

**Authors:** Youfu Liu, Xue Wang, Jiahua Fu, Yanrui Chen, Yan Zhang, Tao Jiang, Zhihua Wang, Na Wang, Lei Zhang

**Affiliations:** 1 Guangxi Key Laboratory of Diabetic Systems Medicine, Guilin Medical University, Guilin, Guangxi, China; 2 School of Humanities and Management, Guilin Medical University, Guilin, Guangxi, China; 3 Guangxi Key Laboratory of Environmental Exposomics and Entire Lifecycle Health, Guilin Medical University, Guilin, Guangxi, China; 4 School of Artificial Intelligence Medicine, Guilin Medical University, Guilin, Guangxi, China

**Keywords:** Andersen’s Behavioral Model, community health service, older adults, geriatric health management, health service utilization, older workers, work–health intersection

## Abstract

**Objective:**

This single-center, cross-sectional study investigates the health management demands, service utilization patterns, and their determinants among the older adults in a community of Liuzhou City, to provide empirical evidence for optimizing community-based older adults health services. With rising retirement ages and the increasing proportion of older workers in China’s labor force, this study also examines how work status and schedule compatibility shape service utilization.

**Methods:**

Using a convenience sampling method, a questionnaire survey was conducted among 320 older adults individuals aged 60 and above in the jurisdiction of a community health service center in Liuzhou City in 2025. Grounded in Andersen’s Behavioral Model, the survey covered predisposing, enabling, and need factors, alongside service utilization. Data were analyzed using descriptive statistics, chi-square tests, and binary logistic regression.

**Results:**

The chronic disease prevalence was 68.4%. While 82.5% were aware of the available community health services, only 45.6% regularly participated. The top three desired services were routine physical examinations (91.2%), chronic disease management (87.5%), and home-based medical services (65.3%). Regression analysis indicated that education level (OR = 2.35, 95% CI: 1.21–4.56), monthly income (OR = 1.98, 95% CI: 1.05–3.73), number of chronic diseases (OR = 3.11, 95% CI: 1.67–5.79), and co-residence with family (OR = 0.45, 95% CI: 0.22–0.92) were significant predictors of active participation. Need factors were the primary driver of participation, while enabling factors acted as critical facilitators or barriers.

**Conclusion:**

A significant “high demand, low utilization” paradox exists. These findings highlight a need for targeted, locally-tailored strategies focusing on vulnerable groups (e.g., low-income, single-living, less-educated older adults) in Liuzhou. Strengthening health education, diversifying services (especially home-based medical services), and integrating family support may enhance the effectiveness of community health management programs. However, further research in diverse settings is required to confirm the generalizability of these conclusions.

## 1 Introduction

The global population is undergoing a profound demographic transition characterized by rapid aging. In China, this phenomenon is particularly acute. By the end of 2023, the population aged 60 and above had reached 297 million, accounting for 21.1% of the total population (1). This surge inevitably leads to a sharp increase in the demand for health services, placing immense pressure on the existing healthcare system (2). Traditional, disease-centered and hospital-based models are no longer sustainable for meeting the multifaceted, long-term, and preventive health needs of the burgeoning older adults demographic (3).

Concurrently, the miniaturization of family units and increased population mobility have weakened traditional family-based care. The “4-2-1” family structure means younger generations face enormous caregiving burdens, and the proportion of empty-nest older adults has surpassed 50% (4). This erosion of traditional support necessitates the urgent exploration of alternative, community-based models of care (5).

Community health services, serving as the foundational tier of the primary healthcare system, possess distinct advantages in older adults health management. These services are geographically proximate, cost-effective, and capable of providing continuous, comprehensive, and coordinated care (6). The Chinese government has mandated a “home-based, community-supported, and institution-supplemented” integrated medical and older adults care service system (7, 8). Within this framework, community health service centers are uniquely positioned to deliver accessible health management interventions (9). With rising retirement ages and the increasing proportion of older workers in China’s labor force, community health services must serve both retired and employed older adults (7). Liuzhou, as an industrial city, has a high rate of post-retirement re-employment, making it a relevant setting to examine work–aging intersections (10). Delayed retirement policies and continued labor market attachment mean that many seniors face time constraints that affect their access to preventive care. This study therefore considers work status as a contextual factor shaping health service utilization (7).

Despite the recognized potential, a significant gap often exists between service availability and actual utilization. Andersen’s Behavioral Model of Health Services Use is a widely accepted paradigm for analyzing healthcare-seeking behaviors, positing that utilization is determined by predisposing, enabling, and need factors (11, 12). However, the relative importance of these factors in the specific cultural and socioeconomic context of Chinese communities remains insufficiently understood, especially regarding proactive health management behaviors (13). This study, therefore, aims to contribute empirical evidence from a typical urban community in Liuzhou City, using Andersen’s model to explore the determinants of health service utilization among the older adults, with the goal of informing local policy and practice.

Therefore, this study aims to fill this research gap by empirically examining the health management demands, service utilization patterns, and their determinants among the older adults in a typical urban community in Liuzhou City. By employing Andersen’s model, this research seeks to disentangle the complex mechanisms through which various factors influence engagement in community health management programs. The ultimate goal is to provide actionable, evidence-based policy recommendations to optimize resource allocation and enhance service delivery for community-dwelling older adults populations, based on an empirical understanding of their current health management needs and utilization patterns.

## 2 Materials and methods

### 2.1 Study design and participants

**Figure 1:**
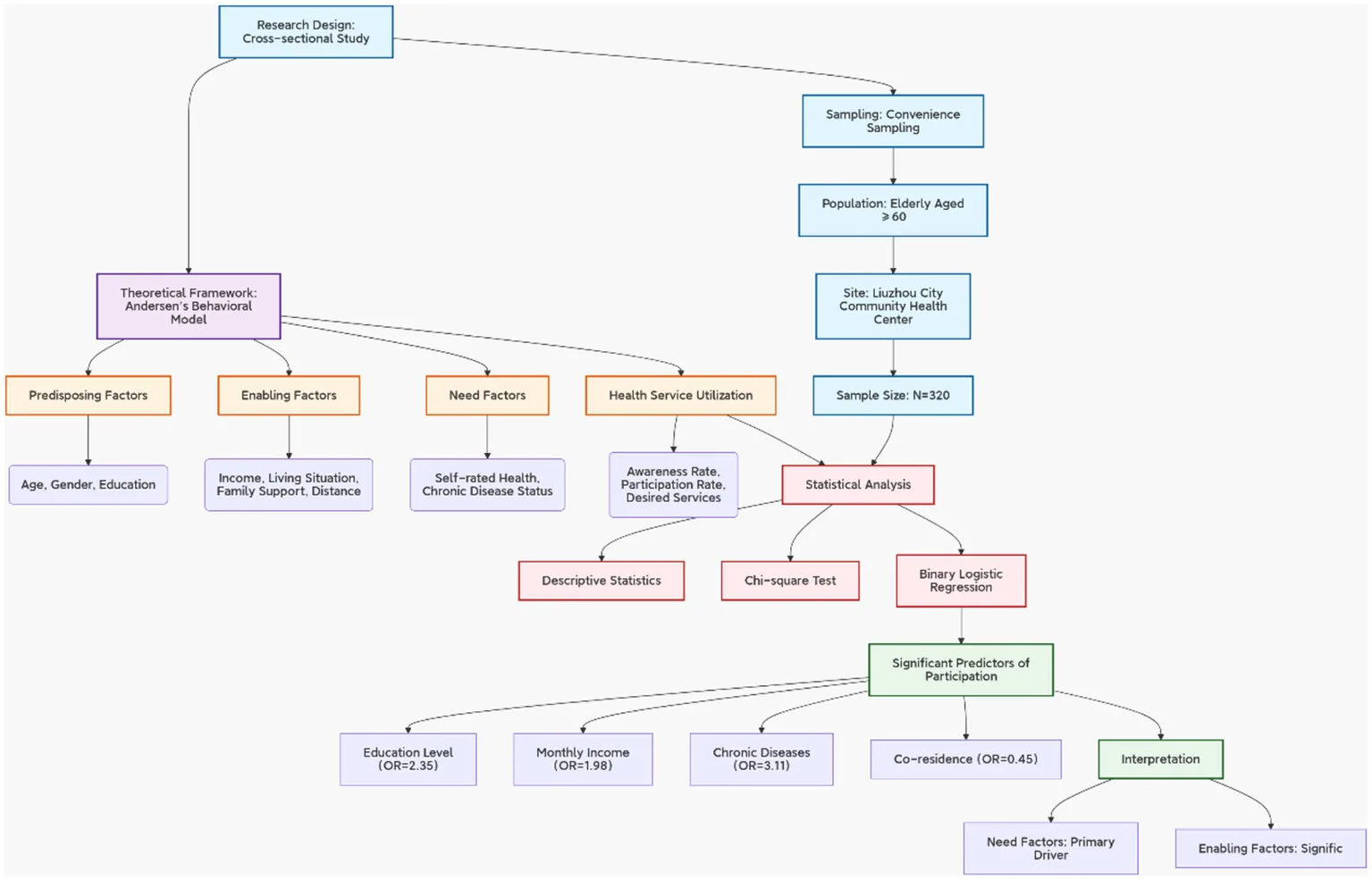
A schematic diagram of the study design and experimental workflow.

This single-center, cross-sectional study was conducted in the jurisdiction of a typical urban Community Health Service Center (CHSC) in Liuzhou City, Guangxi, China, from October to December 2025 (Figure 1). Liuzhou, a major industrial city, has an aging rate slightly above the national average, making it a potentially informative, though not necessarily representative, site for this exploratory investigation (10).

The inclusion criteria were: (1) aged 60 years or older; (2) residing in the community for at least 6 months; (3) possessing basic communication abilities; and (4) voluntarily providing informed consent. Individuals with severe cognitive impairment, psychiatric disorders, or communication barriers were excluded.

Ethical Approval: The study was conducted in accordance with the Declaration of Helsinki. Approval was obtained from the Ethics Committee of Guilin Medical University (Approval No.: GLMC-202410601048). Written informed consent was obtained from all participants prior to their inclusion in the study.

### 2.2 Sampling and sample size

A convenience sampling method was utilized. Data were collected through three primary channels: outpatients visiting the CHSC, follow-up visits by family doctor teams, and centralized surveys at community activity centers. Assuming a significance level (α) of 0.05, a margin of error (δ) of 0.05, and an estimated proportion (P) of 0.5, the minimum required sample size was calculated to be 384 using the formula


$$N=\frac{Z^{2}\times P(1-P)}{\delta^{2}}$$

Anticipating a valid response rate of approximately 70–80%, 450 questionnaires were distributed. Ultimately, 320 valid questionnaires were retrieved, yielding an effective recovery rate of 71.1%. Ultimately, resulting in a sample size slightly below the initial target. This shortfall, primarily due to time and resource constraints in this single-center study, may reduce the statistical power for detecting small effects. Nevertheless, *post hoc* power analysis indicated that the sample size provided over 80% statistical power to detect the moderate-to-large effect sizes (e.g., OR ≥ 2.0) observed in our final multivariate model, supporting the reliability of our main findings.

### 2.3 Measurement and variables

The survey instrument was a self-designed questionnaire, “Questionnaire on the Utilization of Community Older Adults Health Management Services,” developed based on Andersen’s Health Service Utilization Model (14). The development process included: (1) an initial item pool (n = 45 items) generated from an extensive literature review and consultations with a panel of three experts (two geriatric medicine specialists and one public health professor); (2) content validity assessed by the same expert panel. They rated each item for relevance on a 4-point Likert scale. The Scale-level Content Validity Index based on the Universal Agreement method (S-CVI/UA) was 0.91, and the average S-CVI (S-CVI/Ave) was 0.96. The item-level CVI (I-CVI) ranged from 0.83 to 1.00, indicating good relevance at the item level. These results indicated excellent content validity; (3) a pilot test conducted with 50 older adults individuals in a similar community to refine wording and assess reliability (Cronbach’s α = 0.83 for the overall scale, indicating good internal consistency). Each item was explicitly mapped to Andersen’s model to ensure construct validity.

#### 2.3.1 Dependent variable

Regular participation in community health management activities (dichotomous: 0 = No, 1 = Yes). “Yes” was defined as participating in at least one community-organized health management activity (e.g., physical examinations, chronic disease follow-ups, health lectures, family doctor services) per quarter over the past year.

#### 2.3.2 Independent variables

Predisposing Factors: Age (60–69, 70–79, ge 80), gender, education level (junior high school or below, high school or above).

Enabling Factors: Monthly income (<3,000 RMB), living situation (living alone, co-residing with spouse/children), family caregiver support (none, available), distance to the CHSC (<1 km, 1–2 km).

Need Factors: Self-rated health (good, fair, poor), number of chronic diseases (0–1, ge 2), and Activities of Daily Living (ADL) assessed via the Barthel Index (15).

Work-related factors: Although not directly measured in the current survey, employment status (employed, retired, re-employed), occupational history (manual vs. non-manual), and retirement transition stage are recognized as potential determinants. Existing proxies such as income source, age group, and the reported barrier of ‘inconvenient timing’ allow preliminary inference. Future studies should include direct measures of work status.

### 2.4 Data collection and quality control

Trained staff from the CHSC and supervised medical interns administered the questionnaires. To ensure data integrity, investigators received unified training prior to data collection. During the process, on-site supervision was maintained to address queries promptly. Completed questionnaires were double-checked for completeness and logical consistency before being entered into a database by two independent researchers using dual-entry verification to minimize data entry errors.

### 2.5 Statistical analysis

Statistical analyses were performed using SPSS Statistics version 26.0 (IBM Corp., Armonk, NY, United States). Descriptive statistics, including frequencies, percentages, means, and standard deviations, were used to summarize the demographic and health characteristics. Chi-square tests were employed for univariate analysis to examine the associations between each independent variable and the dependent variable. To identify significant predictors of regular participation, a binary logistic regression analysis was performed. In line with Andersen’s Behavioral Model, all theoretically relevant variables across the predisposing, enabling, and need domains were entered simultaneously into the regression model using the Enter method. This approach was chosen *a priori* to adjust for potential confounding effects, regardless of their significance in univariate analyses. The model’s goodness-of-fit was assessed using the Hosmer–Lemeshow test (*χ*^2^ = 7.34, *p* = 0.499) and Nagelkerke’s *R*^2^ (0.24). Multicollinearity was examined using Variance Inflation Factors (VIF); all VIF values were below 2, indicating no significant multicollinearity. The model’s goodness-of-fit was assessed using the Hosmer–Lemeshow test (*χ*^2^ = 7.34, *p* = 0.499) and Nagelkerke’s *R*^2^ (0.24). Multicollinearity was examined using Variance Inflation Factors (VIF); all VIF values were below 2, indicating no significant multicollinearity. The level of statistical significance was set at alpha = 0.05 for all tests.

## 3 Results

### 3.1 Demographic and socioeconomic characteristics

A total of 320 older adults residents were enrolled. As detailed in Table 1, the mean age was 71.6 ± 7.2 years. The majority (83.1%) were aged 60 to 79 years. Females accounted for 52.8%. Regarding education, 74.1% had attained a junior high school education or lower. Socioeconomically, 66.0% reported a monthly income below 3,000 RMB, and 74.4% relied on pensions as their primary source of income. Most participants (96.9%) had children, yet 40.9% lacked a fixed family caregiver, highlighting a potential vulnerability in informal support networks.

**Table 1:** Demographic and socioeconomic characteristics of the participants (*n* = 320).

Variable	Category	*n*	%
Age (years)	60–69	145	45.3
	70–79	121	37.8
	≥80	54	16.9
Gender	Male	151	47.2
	Female	169	52.8
Education Level	Junior high school or below	237	74.1
	High school or above	83	25.9
Monthly Income (RMB)	<3,000	211	66.0
	≥3,000	109	34.0
Living Situation	Living alone	53	16.6
	Co-residing with spouse/children	267	83.4
Fixed Family Caregiver	Yes	189	59.1
	No	131	40.9

Data are presented as frequency (n) and percentage (%).

### 3.2 Health status and healthcare needs

The health profile of the surveyed older adults revealed a high burden of chronic diseases. As shown in Table 2, the overall prevalence of chronic conditions was 68.4%. Hypertension was the most prevalent condition (52.5%), followed by diabetes (27.8%) and arthritis (23.4%). Despite the high morbidity, adherence to regular check-ups among chronic patients was suboptimal (57.5%). Furthermore, 29.1% of the participants exhibited some degree of dependency in their ADL, with 10.9% requiring substantial personal care.

**Table 2:** Health status and expressed service needs of the participants.

Variable	Category	*n*	%
Chronic Disease Status	0 diseases	101	31.6
	1 disease	87	27.2
	≥2 diseases	132	41.2
ADL Independence	Fully independent	227	70.9
	Mildly dependent	58	18.1
	Moderately/Severely dependent	35	11.0
Top Desired Services	Regular health check-ups	292	91.2
	Chronic disease management	280	87.5
	Home-based medical services	209	65.3

Data are presented as frequency (n) and percentage (%). ADL, activities of daily living.

Regarding healthcare demands, there was an overwhelming consensus on the necessity of community-based services. The top three most desired services were routine physical examinations (91.2%), chronic disease management (87.5%), and home-based medical care (65.3%). Over 80% of the respondents expressed a clear, explicit need for enhanced community health interventions.

### 3.3 Utilization of community health services

Although the overall awareness rate of community health services was relatively high at 82.5%, the actual utilization rate was low. Only 45.6% regularly participated in health management activities. The most utilized service was free annual physical examinations (65.3%), while the uptake of other services, such as health lectures (22.5%) and home visits (10.9%), remained critically low.

To further elucidate the barriers to service utilization, participants who rarely or never participated were asked to identify the reasons. As presented in Table 3, the primary barriers were a lack of information (38.5% were unaware of the activities), a perceived lack of need due to feeling healthy (35.1%), and scheduling conflicts with their daily routines (24.1%). Physical immobility (17.8%) and fundamental distrust in the quality of community-level care (13.8%) also posed significant hurdles.

**Table 3:** Primary reasons for low utilization of community health services (*n* = 174).

Reason for non-participation	*n*	%
Unaware of the activities	67	38.5
Felt healthy and saw no need	61	35.1
Inconvenient timing of activities	42	24.1
Physical immobility/inability to travel	31	17.8
Distrust in community health service quality	24	13.8
Already had established healthcare providers (e.g., large hospitals)	19	10.9

The 24.1% reporting ‘inconvenient timing of activities’ is likely concentrated among the young-old (aged 60–69), who constitute 45.3% of the sample and are the cohort most affected by delayed retirement policies and continued labor market attachment.

### 3.4 Determinants of health management participation

Binary logistic regression was performed to identify the significant predictors of regular participation. Variables with *p* < 0.05 in univariate analysis were entered into the model. The model demonstrated good fit (Hosmer–Lemeshow test, chi^2 = 7.34, *p* = 0.499) and an overall prediction accuracy of 73.8%. Multicollinearity diagnostics showed all VIF values were <2, indicating no serious multicollinearity.

As summarized in Table 4, the analysis revealed distinct roles of the three categories of factors:

**Table 4:** Binary logistic regression analysis of factors influencing participation in community health management.

Variable	*β*	S. E.	Wald *χ*^2^	*p*-value	OR	95% CI
Predisposing factors						
Education (Ref: ≤Junior high)						
High school or above	0.855	0.339	6.36	0.012	2.35	1.21–4.56
Enabling factors						
Monthly Income (Ref: <3,000 RMB)						
≥3,000 RMB	0.683	0.322	4.49	0.034	1.98	1.05–3.73
Living situation (Ref: Living alone)						
Co-residing with spouse/children	−0.799	0.359	4.95	0.026	0.45	0.22–0.92
Distance to service site (Ref: ≥2 km)						
<1 km	0.721	0.368	3.84	0.050	2.06	1.00–4.23
Need factors						
Chronic disease status (Ref: 0–1 disease)						
≥2 diseases	1.135	0.328	11.96	0.001	3.11	1.64–5.92
Constant	−2.341	0.527	19.76	<0.001	0.10	–

Need factors were the strongest drivers. Older adults individuals suffering from two or more chronic diseases were over three times more likely to participate compared to those with zero or one disease (OR = 3.11, 95% CI: 1.67–5.79, *p* = 0.001).

Enabling factors acted as significant facilitators or barriers. Higher monthly income (OR = 1.98, 95% CI: 1.05–3.73, *p* = 0.034) and living closer to the CHSC (<1 km) (OR = 2.06, 95% CI: 1.00–4.23, *p* = 0.050) promoted participation. Interestingly, older adults individuals living alone were more likely to engage in these services compared to those co-residing with family (OR = 0.45, 95% CI: 0.22–0.92, *p* = 0.026).

Predisposing factors also played a role. Participants with a high school education or above were 2.35 times more likely to participate than those with lower educational attainment (OR = 2.35, 95% CI: 1.21–4.56, *p* = 0.012).

## 4 Discussion

This study, conducted in a typical urban community in Liuzhou, reveals a critical “high demand, low utilization” paradox in community-based older adults health management. While over 80% expressed a strong need for services-particularly chronic disease monitoring and home-based medical services-less than half actively participated. This disconnect highlights systemic inefficiencies and misalignments between service design and the target population’s needs.

Consistent with Andersen’s Behavioral Model of Health Services Use, our multivariate analysis elucidates the complex interplay of predisposing, enabling, and need factors (16). Need factors, specifically the burden of multiple chronic diseases, emerged as the most potent predictor of service utilization. This aligns with previous literature suggesting that healthcare-seeking behavior in the older adults is predominantly reactive rather than proactive (17). Older adults individuals with more severe health deteriorations are naturally compelled to seek external medical support. However, reliance solely on illness as a catalyst for engagement is suboptimal; ideal health management should be preventive and continuous, aiming to maintain intrinsic capacity before catastrophic decline occurs (18, 19).

A deeper analysis of the barriers reveals critical intervention points. The primary reason for non-participation was a lack of awareness (38.5%), indicating that services are not effectively marketed to the target population. The second most cited reason was “feeling healthy and seeing no need” (35.1%), reflecting a health belief model barrier where elders do not perceive themselves at risk and thus do not engage in preventive care (20). Furthermore, inconvenient timing (24.1%) suggests that services are often scheduled without considering the routines of the older adults or their caregivers. Most glaringly, the overwhelming request for home-based medical services (65.3%) starkly contrasted with its abysmal utilization rate (10.9%). This discrepancy may stem from systemic rigidities identified in other studies, including a shortage of community medical staff, the high time–cost of home visits, and restrictive reimbursement policies that often do not cover domiciliary care (21). While our data did not directly assess these provider-side barriers, the stark contrast between the high demand for and low utilization of home-based services strongly suggests their presence as significant impediments.

**Figure 2:**
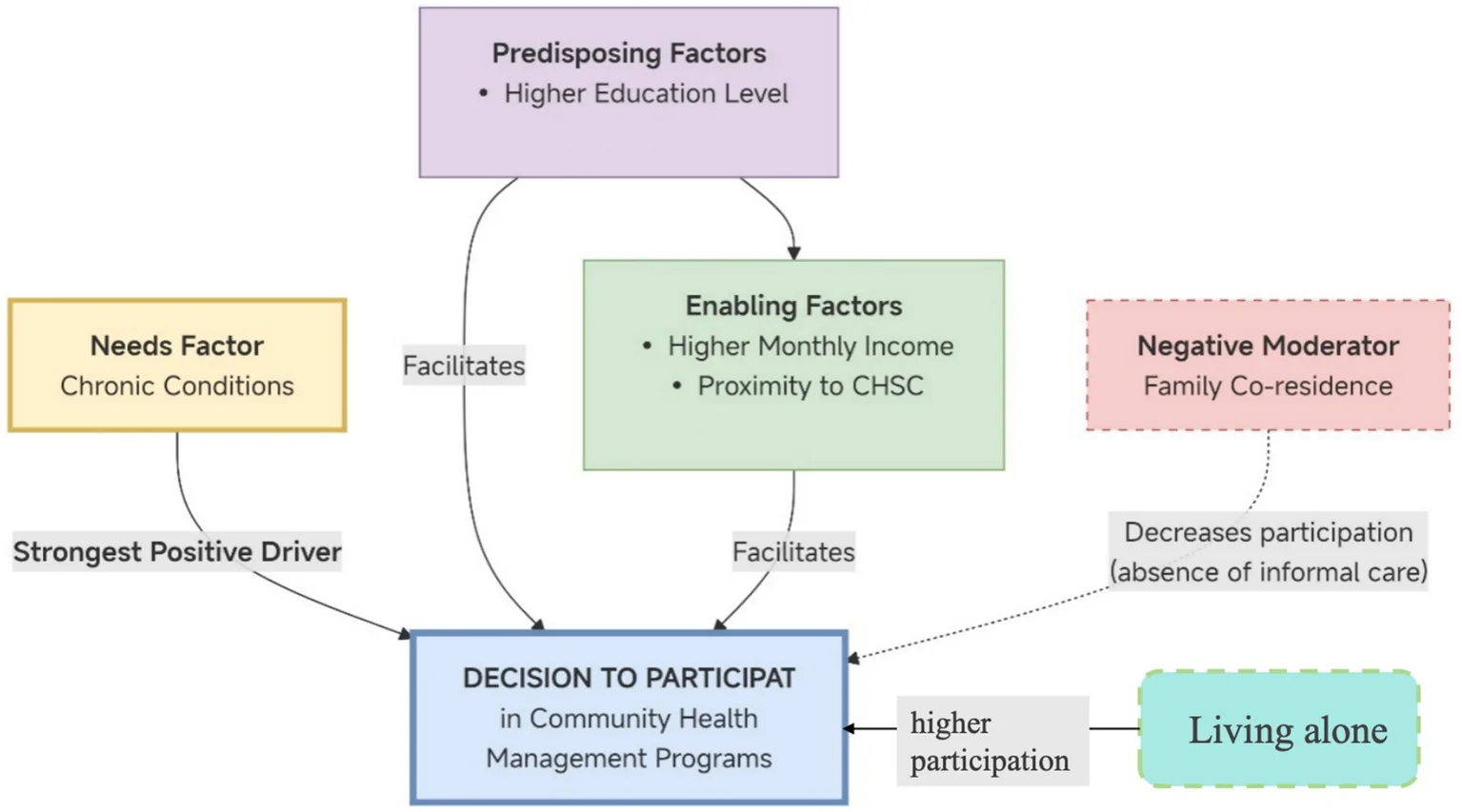
Logical relationship framework of factors influencing older adults health management participation. Family co-residence acts as a negative moderator (decreases participation), while living alone acts as a positive facilitator due to the absence of informal care.

To visually encapsulate these dynamics, Figure 2 synthesizes these relationships, showing how need factors (especially multimorbidity) directly drive participation, while enabling factors such as income and distance moderate access, and predisposing factors like education shape health awareness. Family co-residence showed a negative association, whereas living alone was associated with higher participation, which we discuss as a potential hypothesis related to the absence of informal care.

Beyond the well-documented enabling factors, our findings reveal a critical yet overlooked dimension: the misalignment between community health service schedules and the daily rhythms of older adults who remain economically active. Although China’s statutory retirement age is 60 for men and 55/50 for women, many urban seniors continue working—either through re-employment, informal jobs, or platform-based gigs—driven by financial necessity or personal choice. In our sample, 24.1% of non-participants cited ‘inconvenient timing of activities’ as a primary reason for non-use (Table 3). This proportion is likely concentrated among the ‘young-old’ (aged 60–69), who constitute 45.3% of our respondents and are precisely the cohort most affected by delayed retirement policies and continued labor market attachment. When community health management programs operate exclusively during conventional office hours, they inadvertently exclude older workers who cannot afford to take leave for preventive care. This temporal mismatch mirrors the ‘substitution effect’ we observed for co-residence: just as family support can crowd out formal service use, inflexible service timing crowds out employed seniors. Consequently, the very group that would benefit most from early-stage, work-compatible health interventions—those still productive but facing accumulating chronic risks—becomes systematically underserved. Our results therefore align with the call for ‘age-friendly work environments’ that extend beyond ergonomic adaptations to include flexible, employer-integrated health service delivery. Without explicitly considering work status and schedule compatibility, community health models risk perpetuating inequities between retired and working older adults, undermining the goal of healthy and productive aging.

To bridge these gaps, a paradigm shift is imperative. First, CHSCs must transition from a passive, clinic-based model to a proactive, outreach-oriented approach (26). Leveraging digital health technologies and mobile medical teams can extend the reach of formal healthcare into the homes of the bedridden and isolated (22). Second, health education campaigns must be intensified and culturally tailored to transform the “illness-driven” mindset into a “wellness-driven” culture (23). Finally, recognizing the double-edged sword of family support-where the presence of family caregivers might, in some cases, act as a substitute for formal community services, a phenomenon known as the “substitution effect” (24). Notably, this interpretation is speculative and derived solely from statistical association; it does not imply a causal mechanism, as we did not directly measure caregiving intensity, quality, or family dynamics. This hypothesis requires direct validation in future studies. Future research should explicitly investigate these dynamics. Nonetheless, community programs should proactively involve family caregivers, equipping them with the skills to act as health advocates, thus fostering a potentially synergistic formal-informal care network (25).

### 4.1 Study limitations

This study has several limitations that should be acknowledged. First, the use of convenience sampling from a single Community Health Service Center (CHSC) limits the generalizability of the findings. Our conclusions are primarily exploratory and locally contextualized to an urban industrial city in Southwestern China, and may not be representative of rural areas or other cities. Second, the cross-sectional design precludes causal inferences regarding the observed associations. Longitudinal studies are warranted to track how changes in health status, family dynamics, and policy over time affect service utilization. Third, our data relied entirely on self-reported measures, which are subject to recall bias (e.g., recalling participation frequency) and social desirability bias (e.g., over-reporting service needs or healthy behaviors). Fourth, despite the comprehensive framework, our survey did not include potentially important variables such as cognitive status, mental health (e.g., depression), health literacy, social isolation, digital literacy, and satisfaction with services. Fifth, by excluding individuals with severe cognitive impairment or communication barriers, we may have underestimated the needs and barriers faced by the most vulnerable segment of the older adults population. Finally, the “substitution effect” of family support, while a compelling hypothesis, requires direct measurement in future studies to be confirmed. This temporal exclusion echoes the substitution effect documented by Gong and Hu (24) between family support and formal care. It also reinforces Li et al.’s (27) argument that digital health interventions must be designed around the time constraints of older workers.

## 5 Conclusion

This single-center, cross-sectional study reveals a critical “high demand, low utilization” paradox in community-based older adults health management in Liuzhou City. Need factors (specifically multimorbidity) are the primary drivers of participation, while enabling factors dictate the feasibility of access. Specific barriers, including a lack of awareness, perceived lack of need, inconvenient scheduling, and the underutilization of desired home-based services, are key areas for intervention.

In conclusion, this single-center, cross-sectional study reveals a critical “high demand, low utilization” paradox in community-based older adults health management in the study area. Within this specific context, need factors (specifically multimorbidity) are the primary drivers of participation, while enabling factors dictate the feasibility of access. To optimize health outcomes in comparable Liuzhou communities, local policymakers and health administrators should implement targeted interventions for vulnerable subgroups—including the less educated, low-income, and those living alone. Such initiatives could focus on enriching service portfolios (particularly expanding home-based medical services), strengthening health education, and fostering an integrated support system that bridges family and community resources. Community health centers should offer evening and weekend clinics, deploy mobile health units near industrial parks, and collaborate with employers to conduct workplace-based health screenings for older employees. Such measures directly address the Research Topic’s emphasis on technological innovations and policy adaptations aimed at the inclusion and safety of older adults.

Given the study’s limitations, these findings should be considered hypothesis-generating for future research. Rigorous, multi-center longitudinal studies are urgently needed to validate and extend these findings, and to evaluate the effectiveness of specific interventions designed to overcome the identified barriers.

## Data Availability

The datasets presented in this study can be found in online repositories. The names of the repository/repositories and accession number(s) can be found in the article/Supplementary material.
