# Supplementary material for: Research on the utilization of community health services and its determinants among older adults: a cross-sectional study in Liuzhou City
Source: Front Public Health. 2026 Sep 7;14:1885041. doi: 10.3389/fpubh.2026.1885041 (PMC13590370; doi:10.3389/fpubh.2026.1885041)
Supplement: Supplementary file 1 [file Table_1.DOCX]

**Appendix: Survey Questionnaire**

(Note: The following is the English translation of the original Chinese questionnaire used in the study.)

Title: Questionnaire on Community Elderly Health Management Service Utilization

Dear Elderly Friend,

Hello! In order to understand the health status of the elderly in this community and the demand for community health management services, and to better provide you with health services, we are conducting this survey. This questionnaire is anonymous, and all information will be used for academic research only. We will keep it strictly confidential. Thank you for your support and cooperation!

Instructions: Please tick "√" in the option that matches your situation, or fill in the specific content in the "_". Unless otherwise specified, only one answer is selected for each question. If you have any questions, please consult the investigator.

Part I: Basic Information

1. Your Age: ______ years old

2. Your Gender: (1) Male (2) Female

3. Your Education Level: (1) Illiterate (2) Primary School (3) Junior High (4) High School/Vocational (5) College and above

4. Your Marital Status: (1) Married (with spouse) (2) Widowed (3) Divorced (4) Never married

5. Your Occupation before Retirement: (1) Government/Institution (2) Enterprise/Worker (3) Self-employed (4) Farmer (5) Unemployed (6) Other

Part II: Economic Status

6. Your Current Monthly Income (including pension, allowance, etc.): (1) <1000 RMB (2) 1000-1999 RMB (3) 2000-2999 RMB (4) 3000-3999 RMB (5) 4000-4999 RMB (6) \ge 5000 RMB

7. Your Main Source of Income: (1) Pension (2) Children (3) Savings (4) Government Subsidy (5) Employment (6) Other

8. Your Family's Average Monthly Medical Expenses: (1) <500 RMB (2) 500-999 RMB (3) 1000-1999 RMB (4) 2000-2999 RMB (5) \ge 3000 RMB

9. Your Health Insurance Type: (1) Urban Employee Basic Medical Insurance (2) Urban-Rural Resident Basic Medical Insurance (3) Commercial Health Insurance (4) Public Expense (5) None (6) Other

Part III: Living Situation and Family Support

10. Your Current Living Situation: (1) Alone (2) With spouse only (3) With children (4) With children and grandchildren (5) With other relatives (6) With non-relatives

11. Number of Children: (1) 0 (2) 1 (3) 2 (4) 3 (5) \ge 4

12. Distance to Children: (1) Same household/adjacent (2) Same community (3) Same city, different district (4) Different city (5) No children

13. Fixed Caregiver (multiple choice): (1) Spouse (2) Children (3) Relatives (4) Friends/Neighbors (5) Maid (6) None

14. Caregiver Availability (days/week): (1) <1 day (2) 1-2 days (3) 3-4 days (4) 5-6 days (5) 7 days (6) No caregiver

Part IV: Health Status

15. Your Self-Rated Health: (1) Excellent (2) Good (3) Fair (4) Poor (5) Very Poor

16. Health Change Compared to Last Year: (1) Much better (2) Slightly better (3) Unchanged (4) Slightly worse (5) Much worse

17. Chronic Diseases (multiple choice): (1) Hypertension (2) Diabetes (3) Heart Disease (4) Stroke (5) COPD (6) Arthritis (7) Osteoporosis (8) Tumor (9) Other (10) None

18. Number of Chronic Diseases: (1) 0 (2) 1 (3) 2 (4) 3 (5) \ge 4

19. Medication Adherence: (1) Always (2) Sometimes forget (3) Often forget (4) N/A

20. Regular Check-ups: (1) Yes (2) Occasionally (3) Never (4) N/A

21. ADL Assessment (1=Independent, 2=Needs help, 3=Dependent):

- Feeding (1-3)

- Bathing (1-3)

- Dressing (1-3)

- Toileting (1-3)

- Continence (1-3)

- Transferring/Walking (1-3)

- Stair climbing (1-3)

Part V: Utilization of Community Health Services

22. Awareness of CHSC: (1) Very familiar (2) Somewhat familiar (3) Heard a little (4) Not very familiar (5) Never heard

23. Distance to CHSC: (1) <500m (2) 500m-1km (3) 1-2km (4) 2-3km (5) >3km

24. Used CHSC in the past year? (1) Yes (Go to Q25) (2) No (Skip to Q27)

25. Services Used (multiple choice): (1) Free physical exam (2) Chronic disease follow-up (3) Health lecture (4) TCM service (5) Family doctor (6) Home visit (7) Rehabilitation (8) Psychological counseling (9) Other

26. Frequency of Participation: (1) \ge 1 time/month (2) 1-2 times/quarter (3) 1-2 times/half year (4) \le 1 time/year (5) Never

27. Reasons for Non-participation (multiple choice): (1) Unaware (2) Feel healthy (3) Inconvenient time (4) Mobility issues (5) Distrust (6) Have other doctors (7) Not interested (8) Other

28. Signed a Family Doctor Contract? (1) Yes (2) No (3) Don't know what it is

29. Satisfaction with Family Doctor (if applicable): (1) Very satisfied (2) Satisfied (3) Neutral (4) Unsatisfied (5) Very unsatisfied (6) N/A

Part VI: Health Service Needs and Satisfaction

30. Desired Community Health Services (multiple choice): (1) Regular check-ups (2) Chronic disease management (3) Home visits (4) Health lectures (5) Rehab guidance (6) TCM (7) Psychological counseling (8) Nutrition advice (9) Legal aid (10) Other

31. Level of Need for Services: (1) Very much (2) Somewhat (3) Neutral (4) Not really (5) Not at all

32. Overall Satisfaction with CHSC: (1) Very satisfied (2) Satisfied (3) Neutral (4) Unsatisfied (5) Very unsatisfied (6) Never used

33. Specific Satisfaction Ratings (1=Very unsatisfied, 5=Very satisfied, 0=Never used):

- Service attitude (1-5, 0)

- Technical level (1-5, 0)

- Waiting time (1-5, 0)

- Facilities (1-5, 0)

- Environment (1-5, 0)

- Service variety (1-5, 0)

- Treatment effect (1-5, 0)

- Cost reasonableness (1-5, 0)

34. Aspects Needing Improvement (multiple choice): (1) More services (2) Longer hours (3) Better medical skills (4) Better environment (5) Lower costs (6) More promotion (7) Shorter waiting time (8) Other
